# Microfluidics‐Enabled Nanovesicle Delivers CD47/PD‐L1 Antibodies to Enhance Antitumor Immunity and Reduce Immunotoxicity in Lung Adenocarcinoma

**DOI:** 10.1002/advs.202206213

**Published:** 2023-05-03

**Authors:** Zhenwei Su, Shaowei Dong, Yao Chen, Tuxiong Huang, Bo Qin, Qinhe Yang, Xingyu Jiang, Chang Zou

**Affiliations:** ^1^ Department of Respiratory and Critical Care Medicine, Shenzhen Institute of Respiratory Diseases Department of Clinical Medical Research Center The Second Clinical Medical College of Jinan University, The First Affiliated Hospital of Southern University of Science and Technology Shenzhen People’s Hospital Shenzhen Guangdong 518001 P. R. China; ^2^ Guangdong Provincial Key Laboratory of Advanced Biomaterials Shenzhen Key Laboratory of Smart Healthcare Engineering Department of Biomedical Engineering Southern University of Science and Technology No. 1088 Xueyuan Rd, Nanshan District Shenzhen Guangdong 518055 P. R. China; ^3^ School of Medicine Life and Health Sciences The Chinese University of Hong Kong (Shenzhen) 2001 Longxiang Road Shenzhen Guangdong 518172 P. R. China; ^4^ Department of Hematology and Oncology Shenzhen Children's Hospital 7019 Yitian Road Shenzhen Guangdong 518038 P. R. China; ^5^ Department of Pharmacology and International Cancer Center Shenzhen University Shenzhen Guangdong 518060 P. R. China; ^6^ Aier School of Ophthalmology Central South University Changsha Hunan 410083 P. R. China; ^7^ School of Traditional Chinese Medicine Jinan University Guangzhou Guangdong 510632 P. R. China

**Keywords:** cancer immunotherapy, immune‐related adverse events, microfluidics‐enabled, nanovesicle, ultra‐pH‐sensitive

## Abstract

The CD47/PD‐L1 antibodies combination exhibits durable antitumor immunity but also elicits excessive immune‐related adverse events (IRAEs) caused by the on‐target off‐tumor immunotoxicity, hindering their clinical benefits greatly. Here, a microfluidics‐enabled nanovesicle using ultra‐pH‐sensitive polymer mannose‐poly(carboxybetaine methacrylate)‐poly(hydroxyethyl piperidine methacrylate) (Man‐PCB‐PHEP) is developed to deliver CD47/PD‐L1 antibodies (NCPA) for tumor‐acidity‐activated immunotherapy. The NCPA can specifically release antibodies in acidic environment, thereby stimulating the phagocytosis of bone marrow‐derived macrophages. In mice bearing Lewis lung carcinoma, NCPA shows significantly improved intratumoral CD47/PD‐L1 antibodies accumulation, promoted tumor‐associated macrophages remodeling to antitumoral status, and increased infiltration of dendritic cells and cytotoxic T lymphocytes, resulting in more favorable treatment effect compared to those of free antibodies. Additionally, NCPA also shows less IRAEs, including anemia, pneumonia, hepatitis, and small intestinal inflammation in vivo. Altogether, a potent dual checkpoint blockade immunotherapy utilizing NCPA with enhanced antitumor immunity and reduced IRAEs is demonstrated.

## Graphical abstract

A microfluidics‐enabled nanovesicle using ultra‐pH‐sensitive polymer mannose‐poly(carboxybetaine methacrylate)‐poly(hydroxyethyl piperidine methacrylate) (Man‐PCB‐PHEP) is developed to deliver CD47/PD‐L1 antibodies (NCPA) for tumor‐acidity‐activated immunotherapy. The NCPA can specifically release antibodies in acidic tumor microenvironment, resulting in more favorable treatment effect compared to those of free antibodies. Moreover, NCPA shows less IRAEs, including anemia, pneumonia, hepatitis, and small intestinal inflammation in vivo.

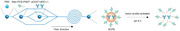

## 1 Introduction

Checkpoint blockade antibody therapy is the hotspot strategy in cancer treatment.^[^ ^1^ ^]^ Recent clinical trials have revealed dual checkpoint blockade immunotherapy would be a more effective candidate to surmount cancer as compared with that of monotherapy, including dual blockade of CD47/PD‐L1.^[^ ^2^ ^]^ Moreover, multiple clinical trials of dual blockade of CD47/PD‐L1 by antibody combinations, bispecific antibodies, or fusion proteins are ongoing, revealing the great potential of this strategy.^[^ ^3^ ^]^ It is noteworthy that CD47/PD‐L1 coexpression predicts long‐term survival and suggests the potential of dual‐targeting immunotherapy in non‐small cell lung cancer.^[^ ^4^ ^]^ However, although accumulated data has shown that the CD47 antibody (aCD47), which blocks the SIRP*α*/CD47 innate immune checkpoint, shows great potential for eliminating tumors by enhancing phagocytic macrophages to phagocytize tumor cells and promote antigen presentation,^[^ ^5^ ^]^ durable antitumor responses to CD47 blockade requires adaptive immune stimulation by a therapeutic antibody such as PD‐L1 antibody (aPD‐L1).^[^ ^6^ ^]^ Nevertheless, the clinical benefit of this strategy is unsatisfactory due to on‐target off‐tumor immunotoxicity^[^ ^7^ ^]^ and the inevitable increased dosage of antibodies also leads to excessive immune‐related adverse events (IRAEs).^[^ ^7^, ^8^ ^]^ Although immunosuppressive drugs, including glucocorticoids, are currently in routine clinical use to manage the toxicity of IRAEs, they induce deleterious outcomes in optimal immunotherapy.^[^ ^9^ ^]^

To tackle these problems, many substantial attempts have been employed, including biomaterial‐delivery platforms for antibody delivery by chemical or biological modification.^[^ ^10^ ^]^ These strategies remain limited clinical transformable so far due to uncontrollable alteration of affinity for antigen binding, uncertain pharmacokinetics, unacceptable stability, and unaffordable costs.^[^ ^11^ ^]^ Thus, to optimize the clinical outcome of dual checkpoint blockade immunotherapy, there is an urgent need to develop a strategy for improving intratumor antibody delivery with reduced IRAEs.

Recently, microfluidics‐enabled synthesis can produce monodisperse particles on a large scale in a straightforward procedure with high controllability and reproducibility, which has been used to produce lipid nanoparticles for the delivery of biomacromolecules, including mRNA and proteins.^[^ ^12^ ^]^ However, the microfluidic synthesis of droplets usually intersects water with organic reagents, such as ethanol and oil, which are harmful to the stability and activity of biomacromolecules and increase tedious purification processes.^[^ ^13^ ^]^ Hence, numerous improvements are needed to enable the production of a delivery platform for biomacromolecules using microfluidic devices.

**Scheme 1:**
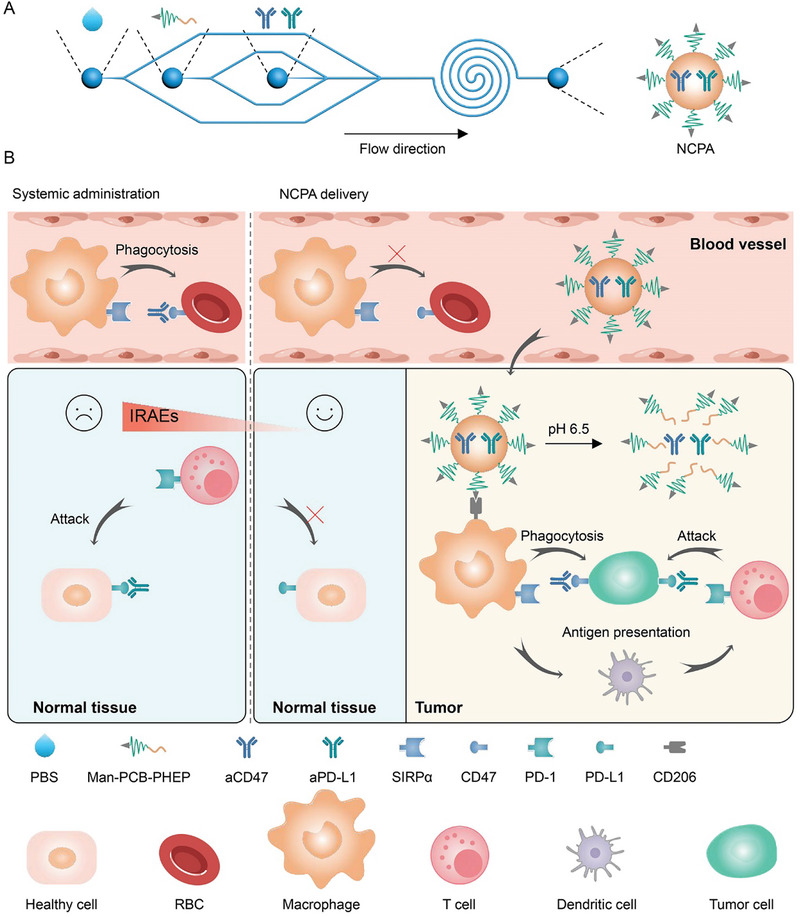
Schematic of microfluidics‐enabled tumor acidity‐responsive nanovesicle to on‐site release CD47/PD‐L1 antibodies for dual checkpoint blockade lung adenocarcinoma immunotherapy with reduced IRAEs. A) Organic solvent‐free microfluidic synthesis of NCPA in one step. B) NCPA on‐site specifically released aCD47 and aPD‐L1 in response to the acidic tumor environment, avoiding IRAEs and inducing synergistic immunotherapy.

In the present study, we demonstrated a microfluidics‐enabled tumor acidity‐responsive nanovesicle to deliver and specifically on‐site release CD47/PD‐L1 antibodies (NCPA) in an acidic tumor microenvironment (TME), thereby assuring dual CD47/PD‐L1 checkpoint blockade and avoiding IRAEs induced by the on‐target off‐tumor immunotoxicity. The NCPA was developed by the organic solvent‐free microfluidic synthesis in one step using ultra‐pH‐sensitive zwitterion polymer Man‐PCB‐PHEP (**Scheme** **1**), maximally preserving the bioactivity of antibodies. Due to encapsulation in mannose‐modified ultra‐pH‐sensitive zwitterion polymeric nanovesicle of NCPA, aCD47 and aPD‐L1 were target‐delivered and specifically on‐site released into the tumor tissue efficiently. Thus, the immune blockade functions of aCD47 and aPD‐L1 were silenced in the blood circulation and healthy tissues but rapidly reactivated in response to acidic TME, thereby reducing IRAEs and inducing robust antitumor immune efficacy in Lewis lung carcinoma (LLC) tumor models by repolarizing tumor‐associated macrophages (TAM) and recruiting dendritic cells (DCs) and cytotoxic T lymphocytes (CTLs). Therefore, the tumor specifically on‐site antibody released by NCPA offers a potential clinically transformable strategy for safely delivering antibody combinations to elicit durable dual checkpoint blockade immunotherapy, which might shed some light on developing a new way for lung cancer treatment.

## 2 Results

### 2.1 Preparation and Characterization of Ultra‐pH‐Sensitive NCPA

**Figure 1:**
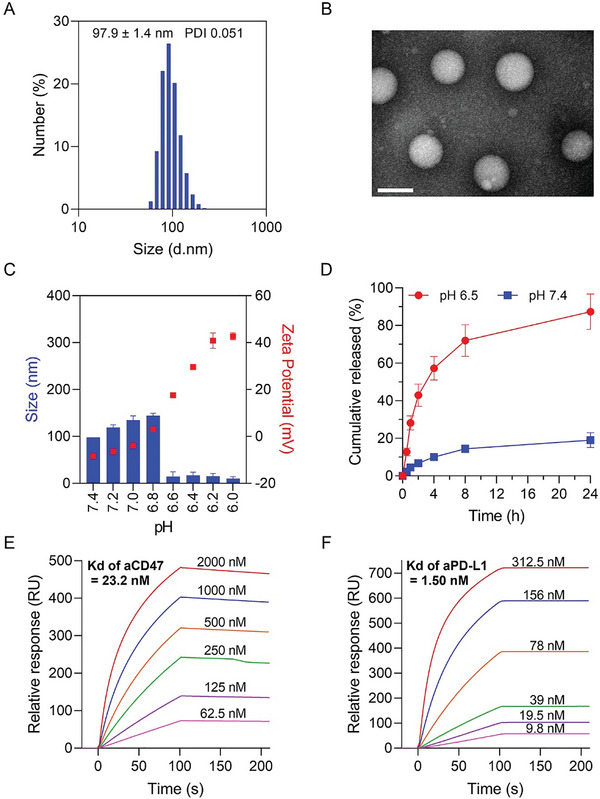
Characterization of NCPA. A) The hydrodynamic diameter of NCPA. B) TEM images of NCPA in pH 7.4. C) Hydrodynamic diameter changes of NCPA in different pH buffers. D) The protein release profiles of NCPA in different pH buffers. E) The in vitro SPR analysis of CD47 antibody binding to CD47 protein. F) The in vitro SPR analysis of PD‐L1 antibody binding to PD‐L1 protein.

First, we synthesized an ultra‐pH‐sensitive zwitterionic polymer, mannose‐poly(carboxybetaine methacrylate)‐poly(hydroxyethyl piperidine methacrylate) (Man‐PCB‐PHEP), by copper‐free organocatalyzed atom transfer radical polymerization of carboxybetaine methacrylate and hydroxyethyl piperidine methacrylate using brominated mannose as the initiator (Scheme S1, Supporting Information). The successful synthesis of Man‐PCB‐PHEP was verified using ^1^H nuclear magnetic resonance (NMR) and gel permeation chromatography (GPC). The characteristic peaks of the synthesized monomers and polymers were labeled in the ^1^H NMR spectrum (Figure S1, Supporting Information), in which the characteristic peaks of the carbon–carbon double bond of the monomers disappeared after polymerization, demonstrating successful synthesis. Moreover, we conducted a GPC analysis of the obtained polymers (Figure S2, Supporting Information), which confirmed that Man‐PCB‐PHEP had a unimodal distribution, ensuring the production of nanodrugs with uniform size. Furthermore, we evaluated the pH responsiveness of Man‐PCB‐PHEP by acid‐base titration. The result demonstrated that Man‐PCB‐PHEP has ultra‐pH sensitivity when the pH was changed from 7.4 to 6.5, owing to the tertiary amino groups of piperidine being completely protonated, resulting in the transformation of Man‐PCB‐PHEP from a hydrophobic state to a hydrophilic state (Figure S3, Supporting Information). Contrastingly, we prepared a multistage microfluidic chip according to an earlier report with modifications for manufacturing NCPA to encapsulate CD47 and PD‐L1 antibodies by the organic solvent‐free microfluidic synthesis in one step. There are three stages and three inlets in the microfluidic chip (Figure S4, Supporting Information). The first stage has a straight channel to introduce a hydrophilic antibody mixture. The inlets of the second and third stages are shunted into two channels for the acid polymer solution and base phosphate buffer and then converge with the first stage at the straight and spiral channels step by step. After optimizing the flow rate ratio of the inlets at these three stages, the monodisperse NCPA was harvested by microfluidics (Figure S5, Supporting Information). The results of the dynamic light scattering (DLS) assay indicated that the diameter of the microfluidics‐enabled nanovesicle was 100.95±1.47 nm and the narrow distribution of polydispersity index (PDI) was 0.117, which showed that it had advantages over nanoprecipitation and double emulsion (Figure S6, Supporting Information). After loading CD47/PD‐L1 antibodies, the size of NCPA was 97.9±1.4 nm without significant changed (**Figure** **1**A). Transmission electron microscopy (TEM) indicated that NCPA had a spherical vesicular structure with a hydrophilic cavity at pH 7.4 (Figure 1B). In contrast, NCPA completely disintegrated at pH 6.5, indicating its ultra‐pH‐sensitivity (Figure S7, Supporting Information). To further test the response to tumor acidity, we incubated NCPA in a series of phosphate buffer solutions with pH values ranging from 7.4 to 6.0. The results demonstrated that NCPA exhibited a significant size change at a narrow pH range of 6.8–6.6, accompanied by a reversal of zeta potential from negative to positive (Figure 1C) due to the protonation of the tertiary amino groups of piperidine groups, resulting in a pH‐sensitive PHEP block that changed from hydrophobic to hydrophilic and the release of negatively charged antibodies. Moreover, quantification of fluorescent‐labeled protein encapsulation efficiency indicated that NCPA fabricated at a rate of 10 mg h^−1^ could encapsulate 94.31±2.78% of protein after optimization (Figure S8, Supporting Information). In this case, the antibody concentration of NCPA was ≈77.9 µg mL^−1^, which was suitable for bedside injection after concentration and aseptic filtration. Therefore, the pH‐responsive release behavior of NCPA demonstrated that less than 20% of the protein was released within 24 h at a pH of 7.4 because they were encapsulated in the lumen of the NCPA (Figure 1D). In contrast, when NCPA was incubated in buffer at a pH of 6.5 mimicking acidic TME, almost 90% of the protein was released within 24 h, apparently because of the decomposition of the vesicular structure of NCPA, as verified by TEM and DLS. The in vitro SPR analysis demonstrated that the dissociation constant (Kd) of the CD47 antibody and PD‐L1 antibody released from NCPA were 23.2 and 1.50 nm, which did not change significantly, indicating the microfluidic synthesis and acidic trigger release would not affect the binding ability of antibody (Figure 1E,F; and Figure S9, Supporting Information). Moreover, while incubating NCPA with bone marrow‐derived macrophages (BMDMs) under observation by confocal laser scanning microscopy (CLSM), it could significantly mount on the cell membrane with reduced phagocytosis (Figure S10, Supporting Information), which was attributed to mannose‐modified NCPA targeted to the mannose receptor CD206 expressed on the outer cell membrane of BMDMs, and the super hydrophilic properties of zwitterion PCB inhibiting cellular uptake. Collectively, we fabricated an NCPA co‐encapsulated with dual immune checkpoint inhibitors of aCD47 and aPD‐L1 by the organic solvent‐free microfluidic synthesis in one step, which exhibited TAM targeting and ultra‐pH sensitivity, demonstrating great potential for antibody delivery.

### 2.2 In Vitro Immune Stimulating Capacity of NCPA

**Figure 2:**
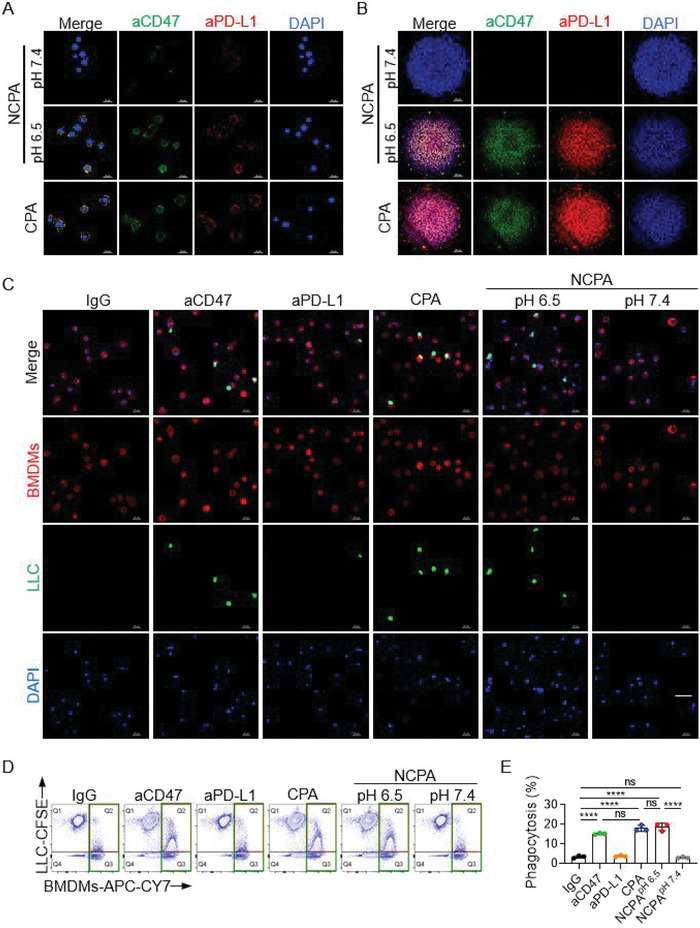
In vitro immune stimulating capacity of NCPA. Confocal images of A) LLC (tumor cells) and B) LLC tumor spheroid cultured with NCPA for 4 h, the aCD47 was stained green with CY3, the aPD‐L1 was stained red with CY5 and the nuclei were stained blue with DAPI. C) Representative CLSM images of LLC (green fluorescently labeled) phagocytosis by BMDMs (red fluorescently labeled) after being treated with IgG, aCD47, aPD‐L1, CPA, and NCPA at pH 7.4 or 6.5. D) Representative flow cytometry counter diagrams showing the phagocytosis of LLC by BMDMs. E) The phagocytosis was calculated as the percentage of double‐positive BMDMs among red fluorescence positive BMDMs, assessed by flow cytometry analysis. Data are presented as mean ± SD (*n* = 3). *p < 0.05; **p < 0.01; ***p < 0.001; ****p < 0.0001.

Blockade SIRP*α*‐CD47 by NCPA could improve the phagocytosis efficiency of LLC cells by repolarized BMDMs. In first, we evaluated the binding affinity of aCD47 and aPD‐L1 released from NCPA. We incubated NCPA with LLC cells after treatment with a buffer at pH levels of 7.4 and 6.5. The weak green fluorescence signal from aCD47 and red fluorescence signal from aPD‐L1 were colocated in the cytoplasm at a pH of 7.4 because the antibodies were coencapsulated in NCPA and slightly uptake by LLC cells (**Figure** **2**A). However, because CD47 and PD‐L1 are expressed mainly on the outer membrane of LLC cells when the pH was changed to 6.5 to induce the release of antibodies, the two kinds of fluorescence signals were separated significantly, as similar as the free CD47/PD‐L1 combination (CPA). The confocal images of LLC tumor 3D spheroid cultured with NCPA after 4 h demonstrated that the fluorescence signal of antibodies appeared only after acidic treatment, which was consistent with 2D culture (Figure 2B). Additionally, NCPA showed negligible hemolysis and cell toxicity, demonstrating good biosafety (Figure S11, Supporting Information). Second, to validate the SIRP*α*‐CD47 immune checkpoint blockade function of NCPA, we investigated the phagocytosis of LLC cells by BMDMs in vitro. The green fluorescence‐labeled LLC cells were treated with various formulations and incubated with red fluorescence‐labeled BMDMs for phagocytosis observation under CLSM (Figure 2C). Compared to the negative control of the IgG group, we observed a significant overlapping fluorescent signal, indicating phagocytosis of LLC cells by BMDMs when treated with the CD47 antibody only, suggesting that the SIRP*α*‐CD47 blockade resulted in enhanced phagocytosis of LLC cells by BMDMs (Figure 2D). On the one hand, the negligible overlapping fluorescent signal when NCPA was treated with BMDMs at pH 7.4, which accounted for the blocking functions of CD47/PD‐L1 antibodies was shielded by the encapsulation of NCPA. On the other hand, due to the acidic pH‐induced responsive cargo release, we found a significantly overlapping fluorescent signal in NCPA at pH 6.5 compared with CD47/PD‐L1 dual blockade treatment. Moreover, quantification of phagocytosis analysis by flow cytometry indicated that more LLC cells were phagocytosed by BMDMs when treated with CD47 mono blockade as compared with the IgG control group (15.22±0.26% vs 3.49±0.54%) (Figure 2D,E). It is noteworthy that the phagocytosis efficiency was further increased to 18.72±1.55% with dual blockade of CD47/PD‐L1 by CPA. In the meantime, after NCPA was incubated with pH 6.5 buffer solution to trigger antibody release, the phagocytosis efficiency of NCPA was determined as 17.98±1.25%, which was consistent with the CLSM observations. Taken together, NCPA was capable of reshaping macrophages to phagocytose LLC cells in vitro.

### 2.3 NCPA Enhances the Intratumor Accumulation of CD47/PD‐L1 Antibodies In Vivo

**Figure 3:**
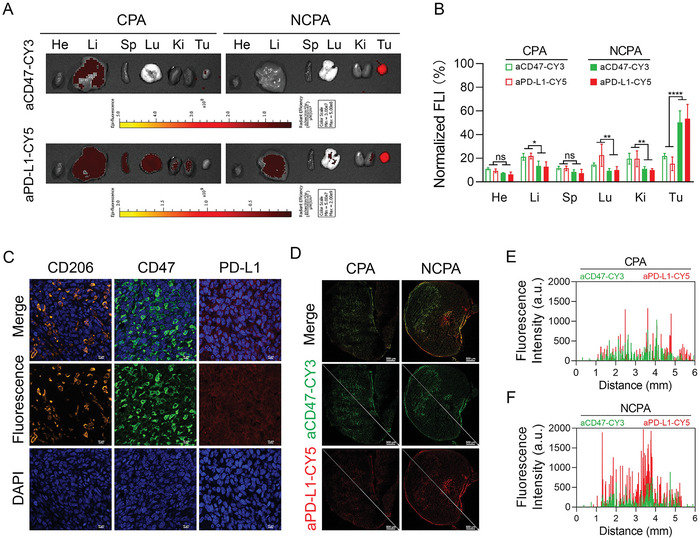
In vivo antibodies delivery efficacy of NCPA. A) Ex vivo fluorescence images of aCD47 and aPD‐L1 in tumors and major organs were collected from mice after 24 h intravenous injection. B) Semi‐quantitative mean fluorescence intensity analysis of ex vivo major organs and tumor. C) Representative images of CD206/CD47/PD‐L1 expression in LLC tissue. D) Confocal images of tumor frozen sections and (E&F) fluorescence intensity analysis of E) free antibody combination CPA and F) NCPA, the aCD47 was stained green with CY3 and the aPD‐L1 was stained red with CY5. *p < 0.05; **p < 0.01; ***p < 0.001; ****p < 0.0001.

However, effective checkpoint blockade immunotherapy depends on high intratumor antibody delivery. Consequently, we evaluated the antibody delivery efficacy of NCPA in vivo. We studied the pharmacokinetics and biodistribution of NCPA in C57BL/6J mice bearing LLC tumor. The pharmacokinetics profiles indicated that NCPA presented longer blood circulation than free aCD47 (1.47±0.03 vs 0.11±0.03 h) and aPD‐L1 (1.51±0.10 vs 0.28±0.16 h) due to zwitterion polymeric nanovesicle shielding protection (Figure S13, Supporting Information). Next, to study the tumor accumulation of NCPA, aCD47 was stained green with CY3, and aPD‐L1 was stained red with CY5. Compared to free antibody administration, antibodies delivered by tumor acidity‐responsive NCPA showed significantly stronger fluorescence signal in the tumor within 24 h in vivo as well as the ex vivo images of the main organ and tumor tissue (**Figure** **3**A; and Figure S14, Supporting Information). Moreover, semi‐quantitative mean fluorescence intensity analysis indicated that free antibodies only accumulated about 20% in the tumor (Figure 3B). In contrast, NCPA delivered about 50% of CD47/PD‐L1 antibodies to the tumor site, indicating an about 2.5‐fold increase in tumor accumulation, which may be ascribed to the mannose‐modified NCPA capable of targeting CD206, which was overexpressed in the lung carcinoma tissue (Figure 3C). Furthermore, because CD47 and PD‐L1 were overexpressed in lung carcinoma tissue (Figure 3C), the images of immunofluorescence sections of tumor tissue indicated that the aCD47 and aPD‐L1 antibodies were bonded to their antigens (Figure 3D). Additionally, fluorescence intensity analysis showed that antibodies penetrated deeper into the tumor tissue by the delivery of NCPA than free antibodies (Figure 3E,F). Therefore, NCPA was capable of target‐delivering aCD47 and aPD‐L1 into a tumor, paving the way for an on‐site specific release of antibodies to induce local antitumor immunotherapy with reduced IRAEs.

### 2.4 NCPA Improves Antitumor Effect Than Free Antibodies In Vivo

**Figure 4:**
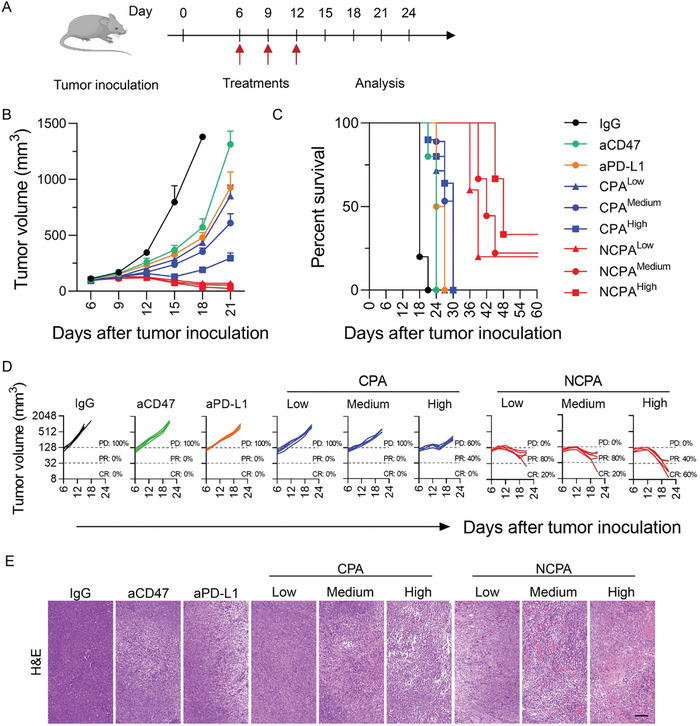
Antitumor growth efficacy of NCPA in subcutaneous graft Lewis lung carcinoma‐bearing mice. A) A schematic illustration showing in vivo administration scheme for Lewis lung carcinoma‐bearing mice after different indication treatments of IgG, aCD47 (100 µg), aPD‐L1 (100 µg), and CP or NCPA at low (50 µg aCD47+50 µg aPD‐L1), medium (100 µg aCD47+100 µg aPD‐L1), and high (200 µg aCD47+200 µg aPD‐L1) dosages (*n* = 5). B) The tumor average volumes. C) The survival plots. D) The trajectory of tumor growth from indicated treatments. PD, progressive disease; PR, partial response; CR, complete response. E) H&E staining images of tumor tissues from mice after different indicated treatments.

Next, we established a subcutaneous LLC model in C57 mice to evaluate the antitumor efficacy of NCPA. When the tumor volume reached 50100 mm^3^, the C57 mice bearing LLC were randomly divided into nine groups and then treated every 3 d for 3 cycles with IgG, aCD47 (100 µg), aPD‐L1 (100 µg), and CPA or NCPA at low (50 µg aCD47+50 µg aPD‐L1), medium (100 µg aCD47+100 µg aPD‐L1), and high (200 µg aCD47+200 µg aPD‐L1) dosages via tail vein injection (**Figure** **4**A). Compared to the exponential growth of the tumor sizes after IgG treatment, tumor growth was significantly inhibited after free antibody treatments of aCD47, aPD‐L1, or CPA (Figure 4B). It is noteworthy that the dual CD47/PD‐L1 blockade treatment induced by free antibodies of CPA demonstrated a dose‐dependent synergistic enhanced effect in tumor growth inhibition due to the deficient tumor accumulation of systemic administration of free antibodies induced by the on‐target off‐tumor immunotoxicity. However, even higher dosage of CPA did not prolong the survival of mice, because only high dosage CPA induced 40% partial response (PD) and 0% complete response (CR) among the free antibody treatments (Figure 4C,D). Therefore, the increase in survival time of mice was not significant after the indicated free antibody treatments (Figure 4C). On the contrary, because of the efficient antibody delivery by NCPA, as described in pharmacokinetic and biodistribution analysis, it showed the most efficient tumor growth inhibition and prolonged mouse survival time among the indicated formulations (Figure 4C). Additionally, with a higher antibody dosage administered by NCPA, tumor growth inhibition and objective responsive rate including PD and CR were further improved, inducing prolonged mouse survival time. Furthermore, the hematoxylin and eosin (H&E) staining images of the tumor sections after the indicated treatments were consistent with the tumor regression effect (Figure 4F). Taken together, NCPA has a potent therapeutic effect by inhibiting tumor growth and extending survival time.

### 2.5 NCPA Elicits Durable Antitumor Immunity In Vivo

**Figure 5:**
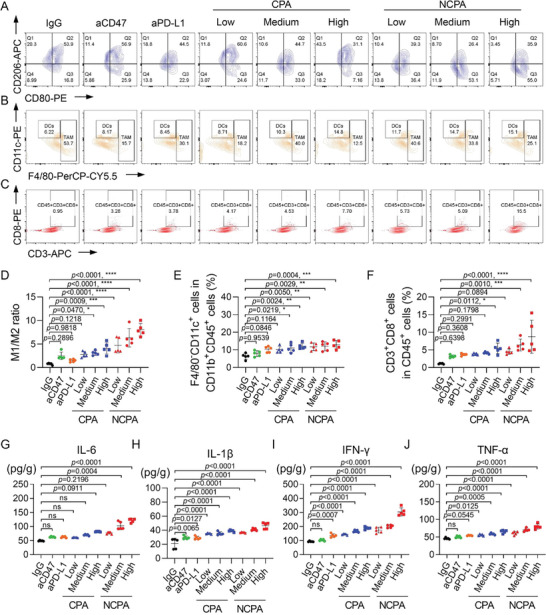
Synergistic antitumor immunotherapy of NCPA in subcutaneous graft Lewis lung carcinoma‐bearing mice. A–C) Representative flow cytometry diagrams of A) TAM, B) DCs, and C) CTLs in tumor tissues after different treatments of IgG, aCD47, aPD‐L1, CPA, and NCPA (*n* = 5). D–F) Quantification analysis of D) CD11b^+^F4/80^+^CD80^+^ M1 macrophages/CD11b^+^F4/80^+^CD206^+^ M2 macrophages ratio. E) CD11b^+^F4/80^−^CD11c^+^ DCs and F) CD3^+^CD8^+^ T cells in tumors by flow cytometry. G–J) ELISA analysis of inflammatory cytokines in tumors, including G) IL‐6, H) IL‐1*β*, I) IFN‐*γ*, and J) TNF‐*α*. *p < 0.05; **p < 0.01; ***p < 0.001; ****p < 0.0001.

Next, to explore the changes in the tumor immune microenvironment induced by NCPA in vivo, the polarization of macrophages and infiltration of DCs and CD8^+^ T cells were analyzed in cell suspensions obtained from tumors treated with various formulations using flow cytometry. The representative flow cytometry counter diagrams and their fraction analysis demonstrated that the M1/M2 ratio was slightly increased after CD47 mono blockade by aCD47 or CD47/PD‐L1 dual blockade by CPA at a low dosage but did not increase the infiltration of F4/80^−^CD11c^+^ DCs and CD3^+^CD8^+^ CTLs (**Figure** **5**A–F). Although the PD‐L1 mono blockade augmented the fractions of F4/80^−^CD11c^+^ DCs and CD3^+^CD8^+^ CTLs without significance, the CD47/PD‐L1 dual blockade by CPA at medium or high dosage induced a significant increase in the M1/M2 ratio and the infiltration of F4/80^−^CD11c^+^ DCs. However, among these free antibody formulations, only a high dose of CPA significantly increased the infiltration of CD3^+^CD8^+^ CTLs. In contrast, NCPA further enhanced the M1/M2 ratio and infiltration of F4/80^−^CD11c^+^ DCs and CD3^+^CD8^+^ CTLs as the dosage increased, which may be ascribed to about 2.5‐fold intratumor accumulation of CD47/PD‐L1 antibodies in comparison with direct intravenous injection. Consistent with the repolarization of TAM from pro‐tumoral M2 to tumoricidal M1 and increasing infiltration of DCs and CTLs, the secretion of intratumoral inflammatory cytokines, including IL‐6, IL‐1*β*, IFN‐*γ*, and TNF‐*α*, was significantly enhanced (Figure 5G–J). Notably, NCPA induced the highest secretion of intratumoral inflammatory cytokines compared to other free antibody formulations. Taken together, with up to 50% intratumor antibodies accumulation, NCPA reversed the immunosuppressive TME to immunoactivated and elicited durable antitumor immunity effectively by dual blockade CD47/PD‐L1.

We have observed resistance to tumor re‐challenge in the NPCA cured mice (Figure S17A, Supporting Information). We have further analyzed the central memory cells in the spleen and tumor‐draining lymphoid node (TDLN). In comparison with the IgG control group, the tumor uncured mice treated with NCPA was only shown a significant increment of central memory (CM) CD8^+^ T cells in TDLN (Figure S17B, Supporting Information). Noteworthily, the tumor cured mice treated with NCPA were shown a significant increment of the whole CD4^+^ and CD8^+^ T cells as well as central memory CD4^+^ and CD8^+^ T cells in both spleen and TDLN, demonstrating the NCPA could potentially elicit immune memory to prevent tumor recurrence.

### 2.6 NCPA Reduces IRAEs Induced by Free CD47/PD‐L1 Antibodies In Vivo

**Figure 6:**
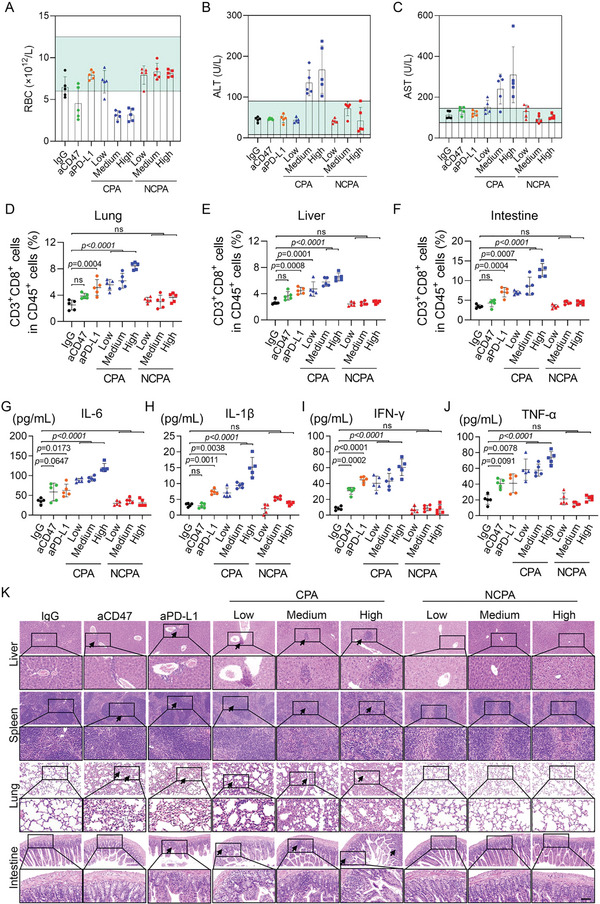
NCPA reduced the incidence of IRAEs in subcutaneous graft Lewis lung carcinoma‐bearing mice. A) Routine blood test results of RBCs (red blood cells) of mice on day 15 after the indicated treatments. (B&C) Blood biochemical analyses of liver function markers. B) ALT (alanine aminotransferase) and C) AST (aspartate aminotransferase) in the serum of mice on day 15 after the indicated treatments. D–F) Flow cytometry analysis of activated CD8^+^ T cell infiltration in D) lung, E) liver, and F) intestine on day 15 after the indicated treatments. G–J) ELISA analysis of inflammatory cytokines in serum, including G) IL‐6, H) IL‐1*β*, I) IFN‐*γ*, and J)TNF‐*α*. K) Ex vivo pathological H&E staining of the liver, spleen, lung, and intestine of mice receiving indicated treatments on day 15. *p < 0.05; **p < 0.01; ***p < 0.001; ****p < 0.0001.

Systemic administration of a free checkpoint antibody would induce unexpected activation of the immune response by increasing the infiltration of lymphocytes in the lung, liver, intestine, and colon, thereby leading to pneumonia, hepatitis, and small intestinal inflammation.^[^ ^7^, ^8^ ^]^ Thus, we evaluated the incidence of IRAEs at the end of drug administration to prove that NCPA could ameliorate the safety profile of checkpoint inhibition therapy. Routine blood tests and blood biochemical tests were performed to assess the body and organ function of the mice. Unsurprisingly, the free aCD47 treatment caused anemia in 60% (3/5) of the mice due to CD47 overexpression on the red blood cell membrane (**Figure** **6**A). However, although PD‐L1 mono blockade did not cause significant dysfunction in hematological tests, the dual CD47/PD‐L1 blockade of CPA not only induced anemia in 100% (5/5) of the mice due to a higher dosage of CD47 antibody administration but also significantly increased the ALT and AST with higher antibody administration (Figure 6A–C). These results revealed the disordered function of the blood and liver in mice induced by systemic administration of free antibodies. In contrast, NCPA did not show significant abnormalities and differences in routine blood or biochemical parameters compared to the IgG control group, demonstrating the enhanced biosafety of NCPA during blood circulation. Moreover, we performed flow cytometry to analyze the infiltration of lymphocytes in the lungs, liver, and intestine. The infiltration of CD45^+^ lymphocytes significantly increased only at medium and high dosages of CPA (Figure S19, Supporting Information), demonstrating that immune homeostasis was disrupted. However, compared to IgG treatment, significant accumulation of CD45^+^CD3^+^CD8^+^ T lymphocytes in the lung, liver, and intestine was induced by PD‐L1 antibody mono‐blockade, and higher infiltration of CD45^+^CD3^+^CD8^+^ T lymphocytes was induced by CPA as the dosage increased (Figure 6D–F). Similarly, systemic administration of free antibodies triggered cytokine release syndrome, which induced the evaluated secretion of IL‐6, IL‐1*β*, IFN‐*γ*, and TNF‐*α* with increasing concentrations of antibody administration (Figure 6G–J). However, the antibodies delivered by our NCPA platform did not induce significant cytokine release syndrome (Figure 6D–J). Furthermore, we performed histological and immunohistochemical (IHC) studies of tissue sections from the main organs to further evaluate the incidence of IRAEs. H&E staining images demonstrated that treatment with free antibodies resulted in detectable inflammatory neutrophil infiltration (Figure 6K). Consistent with H&E staining, IHC results indicated a significant increase in the proinflammatory cytokine IFN‐*γ* in the liver, spleen, and lung of mice treated with free antibodies (Figure S21, Supporting Information). However, no significant lesions or IFN‐*γ* expression were found in the main organs of mice treated with NCPA at different dosages in comparison with the IgG treatment, suggesting the amelioration of systemic IRAEs. Therefore, these results revealed that the incidence of IRAEs of systemic administration immune checkpoint inhibitor combinations of aCD47 and aPD‐L1 was significantly reduced by NCPA.

## 3 Discussion and Conclusion

Increasing experimental evidence and clinical practices are encouraged to evaluate the synergistic tumor remission of dual CD47/PD‐L1 checkpoints blockade immunotherapy. However, the balance of objective response rate and prolonged survival induced by systemic administration of free checkpoint antibodies combination is still unsatisfactory from immunosuppressed tumor microenvironment and on‐target off‐tumor immunotoxicity. Currently, engineering strategies, including fusion protein masking,^[^ ^14^ ^]^ bispecific antibodies,^[^ ^15^ ^]^ and biomaterial‐based delivery,^[^ ^10^, ^16^ ^]^ have been increasingly focused on reducing IRAEs while maintaining durable antitumor immunity in mono or dual checkpoint blockade immunotherapy. However, the clinical practice of these strategies has been largely limited by uncontrolled alteration of binding affinity, uncertain pharmacokinetics, and unaffordable cost.^[^ ^10^, ^11^ ^]^ Here, we developed a tumor acidity‐responsive nanovesicle for codelivery and on‐site release of CD47/PD‐L1 antibodies, which was engineered by the organic solvent‐free microfluidic synthesis in one step using ultra‐pH‐sensitive polymer Man‐PCB‐PHEP. The application of optimally sized, CD206 targeted, and ultra‐pH‐sensitive zwitterionic vesicular NCPA for CD47 and PD‐L1 antibody codelivery helped to increase about 2.5‐fold accumulation of CD47/PD‐L1 antibodies in tumors rather than free antibody after systemic administration. By encapsulating CD47/PD‐L1 antibodies into the ultra‐pH‐sensitive polymeric nanovesicle of NCPA, they can be shielded from normal tissues in circulation and extracellular released only after nanovesicle dissociation upon tumor acidic environment, thereafter ameliorating the IRAEs, including anemia, pneumonia, hepatitis, and small intestinal inflammation induced by on‐target off‐tumor effect and enabling tumor on‐site specific activation immunotherapy, suggesting that NCPA could be a potential clinical candidate for safe and effective dual checkpoint blockade immunotherapy using antibody combinations.

Biomaterial platforms have been increasingly studied to deliver checkpoint antibodies in recent years and obtained fantastic efficacy.^[^ ^10^ ^]^ But what cannot be ignored is that mounted antibody on the platform by the biochemistry modification has low encapsulated efficiency and changes the conformation, binding affinity, and immunogenicity of the antibody, resulting in potential risk in pharmacological activity and safety.^[^ ^10a,d^ ^]^ In comparison to time‐consuming and expensive new drug research and development of biomaterials incorporated delivery platform engineered by traditional batch synthesis, our microfluidics‐enabled easy‐to‐fabricate NCPA is a new pharmaceutical dosage form, which has realized 94.31±2.78% protein encapsulation efficiency and avoided the uncontrollable biochemistry modification and tedious purification.^[^ ^13^ ^]^

It should be noted that TAMs occupied a major sub‐population in the TME, and the reversal of TAMs polarization to tumoricidal M1‐like by SIRP*α*/CD47 blockade has shown the potential to synergize with PD‐1/PD‐L1 blockade immunotherapy in preclinical studies and ongoing clinical trials.^[^ ^3^, ^6^, ^17^ ^]^ However, the therapeutic outcome of dual checkpoint blockade is hindered by insufficient intra‐tumor antibody delivery. Our NCPA accumulated in the tumor efficiently to reprogram the TAM and promote DCs maturation, thereby inducing robust antigen presentation and enhancing infiltration and antitumor immunity of CD8^+^ T cells to boost reinforced CD47/PD‐L1 dual blockade combination immunotherapy, leading to enhanced tumor remission and extended animal survival.

Regarding the potential for clinical translation in the future, our microfluidics‐enabled easy‐to‐fabricate NCPA reduced the IRAEs significantly while maintaining dual checkpoint blockade immunotherapy, showing unparalleled advantages compared with the promising CD47/PD‐L1 fusion protein or bispecific antibody. Because the combination antibodies are mixed before the microfluidics‐enabled fabrication, it is generally an alternative to other immunomodulators, including checkpoint antibodies, cytokines, agonists, and adjuvants. We envision that the strategy demonstrated in this study could be generalized to deliver other immunomodulator combinations wherein tumor site‐specific immune activation is limited. Moreover, NCPA is a new pharmaceutical formulation instead of a new drug, which is favorable for rapid clinical translation. However, NCPA should be carefully evaluated as an investigational new drug (IND), enabling good laboratory practice (GLP) toxicology studies to confirm its safety before application in human research.

## 4 Experimental Section

### Preparation of NCPA by the Organic Solvent‐Free Microfluidic Synthesis in One Step

To avoid using organic solvents, which may be harmful to the bioactivity of antibodies, the primitive aqueous solution was introduced by syringe pumps (PHD Ultra, Harvard Apparatus). First, Man‐PCB‐PHEP was dissolved in a hydrochloric acid solution of pH 4 at a concentration of 4 mg mL^−1^. Then, the antibody solution (10 mg mL^−1^) was introduced into the chip through the center inlet 1 at 0.2 mL h^−1^. And inlet 2 introduced polymer hydrochloric acid solution (4 mg mL^−1^) at 4 mL h^−1^, and inlet 3 introduced a base sodium hydroxide solution (pH 8) at 40 mL h^−1^. At last, the collected solution from the outlet was filtrated by a 220 nm syringe filter and stored at 4 °C for further usage.

### Antibody Loading Content and pH‐Responsive Release Behavior of NCPA

First, BSA as the substitute for antibody, labeled with CY5 if needed, was encapsulated into NCPA using the microfluidic chip. Then, the collected solution was treated with PBS at pH 7.4 and 6.5 for 0.5, 1, 2, 4, 8, and 24 h and ultracentrifugated by an Amicon centrifugation unit (MWCO, 300 kDa). Finally, the fluorescent signal was recorded by fluorescence spectrometer and the protein concentration was quantified by Pierce BCA Protein Assay Kit (Thermo Scientific) as manufacture instructions.

### LLC Cells Phagocytosis by BMDMs

The BMDMs were seeded to a 6‐well plate at a concentration of 4×10^5^ cells per well and cultured with DMEM overnight. Then, the BMDMs were stained with CD45‐APC‐CY7 and cultured with RPMI‐1640. The LLC cells were treated with IgG, aCD47, aPD‐L1, CPA, and NCPA (pH 7.4 and 6.5) for 1 h and then stained with CFSE by 10 min incubation at room temperature. After repeating freeze‐thaw cycles 3 times with liquid nitrogen and washing with PBS 3 times, the CFSE labeled LLC cells were cocultured with BMDMs in 6‐well plates at a concentration of 1×10^5^ cells per well for 2 h. After aspirating the dissociative LLC cells, the nuclei of reserved cells were stained with DAPI after fixing by 4% paraformaldehyde and captured the images by confocal laser scanning microscopy. Similarly, the coculture cells were collected without aspirated and analyzed by BD FACSCanto Clinical Flow Cytometry System.

### Animal Model

Animal experiments were approved by the Second Clinical Medicine College of Jinan University (AUP‐220302‐SZW‐029‐01). Mouse transportation, housing, and breeding were conducted according to the recommendations of “The use of nonhuman animals in research.” Mice were killed by cervical dislocation to prevent suffering. All mouse experiments were conducted by the ethical standards and guidelines of the Asian Federation of Laboratory Animal Science Associations.

To construct the xenografted LLC mouse tumor model, a total of 5×10^5^ cells suspended in PBS were subcutaneous injected into the right flank of 4 weeks old C57BL/6J male mice. When the tumor volume reached 50–100 mm^3^ after about 1 week of inoculation, the mice were randomly divided for designed experiments.

### In Vivo and Ex Vivo Fluorescence Imaging

LLC‐bearing mice were administrated with CPA or NCPA at an antibody concentration of 200 µg per mouse, while the aCD47 was stained with CY3 and aPD‐L1 was stained with CY5. The in vivo animal fluorescence imaging was conducted at predetermined time intervals (0, 2, 4, 8, and 24 h after the injection of nanomedicine) using the IVIS imaging system, and the ex vivo imaging was performed after removing organs and tumors from mice. Moreover, frozen sections of tumor tissues were captured by CLSM.

### Evaluation of Antitumor Efficacy

The LLC‐bearing mice were randomly divided into 9 groups (*n* = 5) and administrated with IgG, aCD47 (100 µg), aPD‐L1 (100 µg), and CPA or NCPA at low (50 µg aCD47+50 µg aPD‐L1), medium (100 µg aCD47+100 µg aPD‐L1), and high (200 µg aCD47+200 µg aPD‐L1) dosages via tail vein injection at day 6, 9, and 12, respectively. The body weight and tumor volume were recorded every 3 days. The tumor volume was obtained by the formula tumor volume = (width^2^ × Length)/2 for caliper measurements. On day 15 or 21, blood, organs, and tumors were collected for other experiments.

### Statistics

All the data are expressed as mean ± standard deviation (SD). Statistical differences were performed using a one‐way analysis of variance (ANOVA) followed by Tukey's test with GraphPad Prism 9.0 software (GraphPad Software Inc, USA). Tukey's post hoc tests and one‐way analysis of variance (ANOVA) were performed for multiple comparisons. *p* < 0.05 was considered statistically significant.

## Conflict of Interest

The authors declare no conflict of interest.

## Supporting information

Supporting Information

## Data Availability

The data that support the findings of this study are available from the corresponding author upon reasonable request.
