# Supplementary material for: Microfluidics‐Enabled Nanovesicle Delivers CD47/PD‐L1 Antibodies to Enhance Antitumor Immunity and Reduce Immunotoxicity in Lung Adenocarcinoma
Source: Adv Sci (Weinh). 2023 May 3;10(20):2206213. doi: 10.1002/advs.202206213 (PMC10369263; doi:10.1002/advs.202206213)
Supplement: Supplementary file 1 — Supporting Information [file ADVS-10-2206213-s001.pdf]

ADVANCED SCIENCE
Open Access

Supporting Information

for *Adv. Sci.*, DOI 10.1002/advs.202206213

Microfluidics-Enabled Nanovesicle Delivers CD47/PD-L1 Antibodies to Enhance Antitumor Immunity and Reduce Immunotoxicity in Lung Adenocarcinoma

Zhenwei Su, Shaowei Dong, Yao Chen, Tuxiong Huang, Bo Qin, Qinhe Yang, Xingyu Jiang and Chang Zou**

Microfluidics-enabled nano-vesicle delivers CD47/PD-L1 antibodies to enhance anti-tumor immunity and reduce immunotoxicity in lung adenocarcinoma

Zhenwei Su†, Shaowei Dong†, Yao Chen, Tuxiong Huang, Bo Qin, Qinhe Yang, Xingyu Jiang, and Chang Zou**

Materials and Methods

Materials and reagents

The mannose and dihydrophenazine PC™ B0301 were purchased from Sigma-Aldrich. The 2-bromoisobutyryl bromide was obtained from Tokyo Chemical Industry (Shanghai). The methacryloyl chloride and 2-hydroxyethyl piperidine were purchased from Alfa Aesar. CY3 and CY5 NHS ester were purchased from Dalian Meilun Biotech. Co., Ltd (Dalian, China). The other reagents were analytical or chromatogram grade and used as received. The DMEM culture medium, RPMI-1640 culture medium, PBS, 0.25% trypsin-EDTA, and fetal bovine serum (FBS) were purchased from Thermo Fisher Scientific (Gibco, USA). The monoclonal antibodies, including IgG, aCD47, and aPD-L1 used *in vitro* and *in vivo* were purchased from Bioxcell (Ohio, USA). The antibodies used in flow cytometry analysis, including CD45, CD11b, F4/80, CD11c, CD80, CD206, CD3, CD4, CD8a, anti-IFN-γ, and anti-granzyme B, were purchased from Biolegend (USA). The ELISA kits for aCD47 and aPD-L1 were purchased from MEIMIAN (Jiangsu, China). The ELISA kits for IL-6, IL-1β, IFN-γ, and TNF-α were purchased from Elabscience (Wuhan, China). Secondary antibodies for immunofluorescence and immunohistochemical staining include goat anti-rabbit IgG H&L (Alexa Fluor® 488), goat anti-rabbit IgG H&L (Alexa Fluor® 555), goat anti-rabbit IgG H&L (Alexa Fluor® 647), and goat anti-rabbit IgG H&L (HRP) antibody were purchased from Abcam.

Synthesis of mannose-bromide (Man-Br)

The D-mannose-bromide (Man-Br) was synthesized according to patent

WO2014199174A1[1]. Briefly, D-mannose (4.5 g, 2.5 mmol) was dissolved by anhydrous tetrahydrofuran (20 mL) in a Schlenk flask equipped with a magnetic stirrer and the solution was bubbled by N_2 for 10 min. After adding triethylamine into the flask, 2-bromoisobutyryl bromide (3.15 mL, 25 mmol) was transferred to the flask drop-wise to perform the reaction under N_2 protection for 24 h. The mixture was dropped into an excess amount of cold petroleum ether to precipitate. After filtering and washing with petroleum ether, the solid was re-dissolved in chloroform and precipitated by cold petroleum ether twice. The solid was dried by vacuum to yield a white waxy product. (7.09 g, 86.2%)

Synthesis of 2-hydroxyethyl piperidine methacrylate

A well-stirred, ice-cooled solution of 2-hydroxyethyl piperidine (1.32 mL, 10 mmol) in an aqueous sodium hydroxide (1.60 g, 20 mmol, 25 mL) was treated with dropwise addition of methacryloyl chloride (1.19 mL, 11 mmol) over a period of ~15 min. After 30 min of stirring on ice, the reaction mixture was allowed to reach room temperature and stirred for a further 4 h. The mixture was adjusted to pH 7 using dilute HCl (0.1 M). The resulting beige mixture was extracted with dichloromethane (DCM) and washed 6 times with NaCl saturated solution. The DCM solution was dried with anhydrous sodium sulfate over 0.5 h. After filtration, DCM was removed by evaporating to obtain a light yellow liquid product.

Synthesis of mannose-poly (carboxybetaine methacrylate) (Man-PCB)

The copolymer Man-PCB was synthesized by copper-free organic ATRP according to literature[2]. A 10 mL schlenk tube charged with a magnetic stirrer was added with initiator Man-Br (16.5 mg), catalyst dihydrophenazine PC™ B0301 (0.5 mg), monomer carboxybetaine methacrylate (200 mg), and solvent dimethylformamide (DMF) (10 mL). The tube was sealed and degassed by freeze-thawing three times. After 24 h reaction under white LED light, the mixture was dialysis against deionized water. The resulting solution was lyophilized to obtain a white product.

Synthesis of mannose-poly(carboxybetaine methacrylate)-poly(2-hydroxyethyl piperidine methacrylate) (Man-PCB-PHEP)

50 mg Man-PCB was dissolved by DMF in a 50 mL schlenk tube charged with a

magnetic stirrer. Then, the catalyst dihydrophenazine PC™ B0301 (0.5 mg) and monomer HEPMA (2.50 g) were dissolved in DMF (10 mL) and transferred into the tube. The tube was sealed and degassed by freeze-thawing three times to process 24 h reaction under a white LED light. Afterward, the mixture was adjusted to pH 5 using dilute HCl (0.1 M) and dialysis against deionized water. The resulting beige solution was lyophilized to obtain a white yellow product.

Design and fabrication of microfluidic chip

The structure of the microfluidic chip for tumor-acidity responsive NCPA synthesis is shown in Figure S5, which has three inlets, two prismatic-ambulatory-plane channels, two straight mixing channels, one 5 loops double spiral mixing channel, and one outlet. And the channel of this chip was 100 μm wide and 60 μm depth. The master mold and the chip were fabricated as previously described.

Characterization of synthetic polymers

^{1}H NMR spectra were obtained using an AVANCE III 400M spectrometer. D_2O or $CDCl_3$ was used as the solvent depending on their solubility. The molecular weight and molecular weight distribution of the polymers were estimated using a gel permeation chromatography (GPC) system in the water phase. The pKa of Man-PCB-PHEP was evaluated by titration according to the literature[3].

Physicochemical characterization of NCPA

The hydrodynamic sizes and zeta potentials of NCPA were determined by dynamic light scattering (DLS). Measurements were performed at 25 °C using the Malvern Zetasizer Nano ZS instrument (Malvern Instruments Ltd, UK). The data of particle sizes and zeta potentials were collected on an auto-correlator with detection angles of scattered light at 175°, respectively. Transmission electron microscopy (TEM) was performed using an HT7700 operated at 120 kV. The samples were prepared by drying a drop (6 μL, 1 mg/mL) of the sample solution on a copper grid coated with amorphous carbon. For the negative staining of samples, a small drop of uranyl acetate solution (1 wt% in water) was added to the copper grid, which was then blotted with a filter paper after 1 min. Finally, the grid was dried overnight in a desiccator before TEM observation.

Bone marrow isolation and Bone marrow-derived macrophages (BMDMs) culture

Progenitor cells were isolated from murine bone marrow and cultured in DMEM with M-GSF (20 ng/mL) according to methods described in the literature[4]. Briefly, the collected progenitor cells from the bone marrow of a mouse were seeded in a T75 cell culture flask. On day 3, media was aspirated and washed with PBS once and replaced with fresh DMEM with M-GSF (20 ng/mL). On day 7, the cells were dislodged by trypsin digestion in 10 minutes and counted with a hemocytometer for further experiments.

Isolation and culture of splenocytes

Splenocytes were isolated from the murine spleen and cultured in RPMI-1640 with IL-2 (20 ng/mL) according to methods described in the literature[5]. The collected splenocytes cells from a mouse spleen were seeded in a T75 cell culture flask. The media was replaced with fresh RPMI-1640 supplemented IL-2 (20 ng/mL) every two days. On day 7, the matured T cells were counted with a hemocytometer for further experiments.

Cell culture

Lewis lung carcinoma cells were cultured in Dulbecco's modification of Eagle medium (DMEM) supplemented with 10% (vol/vol) FBS, 1% L-glutamine, and 1% penicillin and streptomycin (P/S). And all cells were maintained at 37 °C and 5% CO_2.

Tumor spheroid

The 3D tumor spheroid was established according to literature[6]. Briefly, target cells (LLC) were seeded 5,000 cells per well in an ultra-low attachment 96-well round bottom microtiter plate to generate 3D tumor spheroid (24-48h). On the one hand, tumor spheroid was incubated with fluorescence labeled NCPA to evaluate the acidic responsive release. On the other hand, the spheroid was used to compare phagocytosis efficiency of CPA and NCPA. After the spheroids were formed, the target cells (100,000) were stained with CFSE prior incubating with NCPA for 30 min and incubating with effector cells (RAW 264.7) labeled with anti-CD45 CY7 antibody, the spheroids were observed under a confocal microscope after fixing with PFA and staining with DAPI or analyzed by flow cytometry.

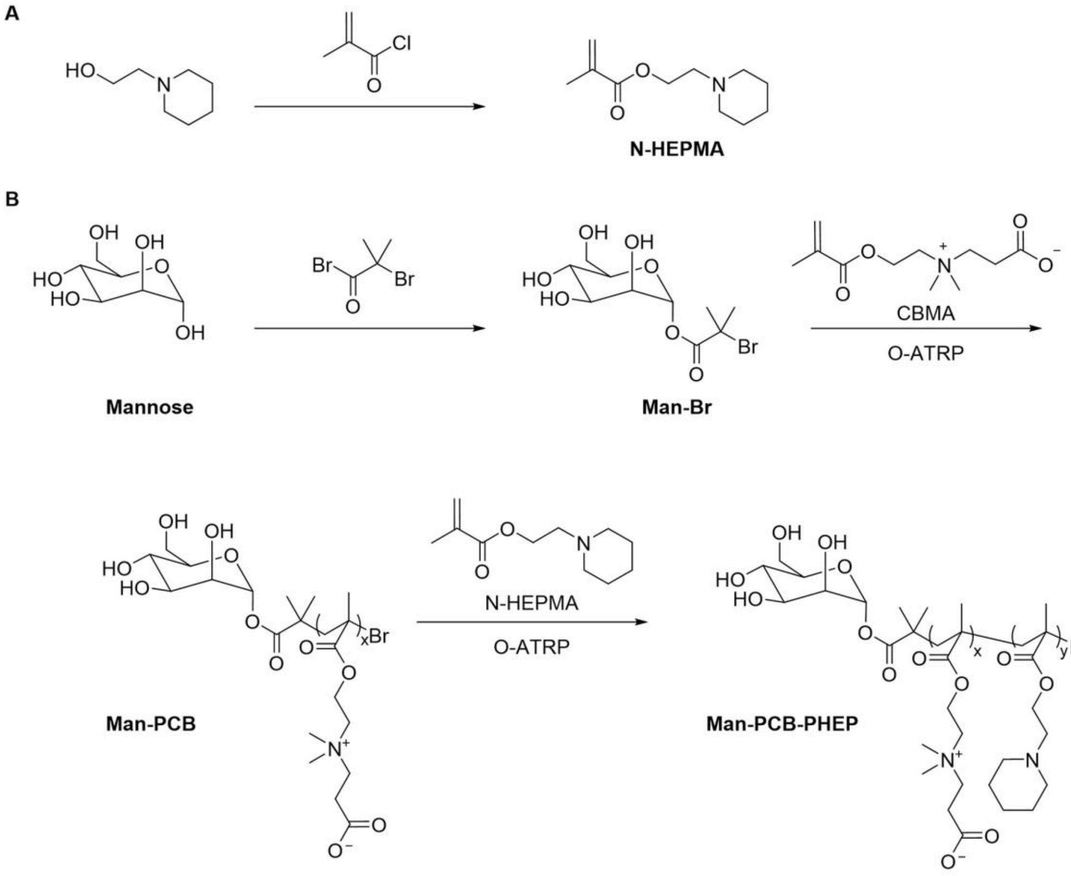

Scheme S1. Schematic synthesis routine of ultra pH-sensitive zwitterionic polymer Man-PCB-PHEP.

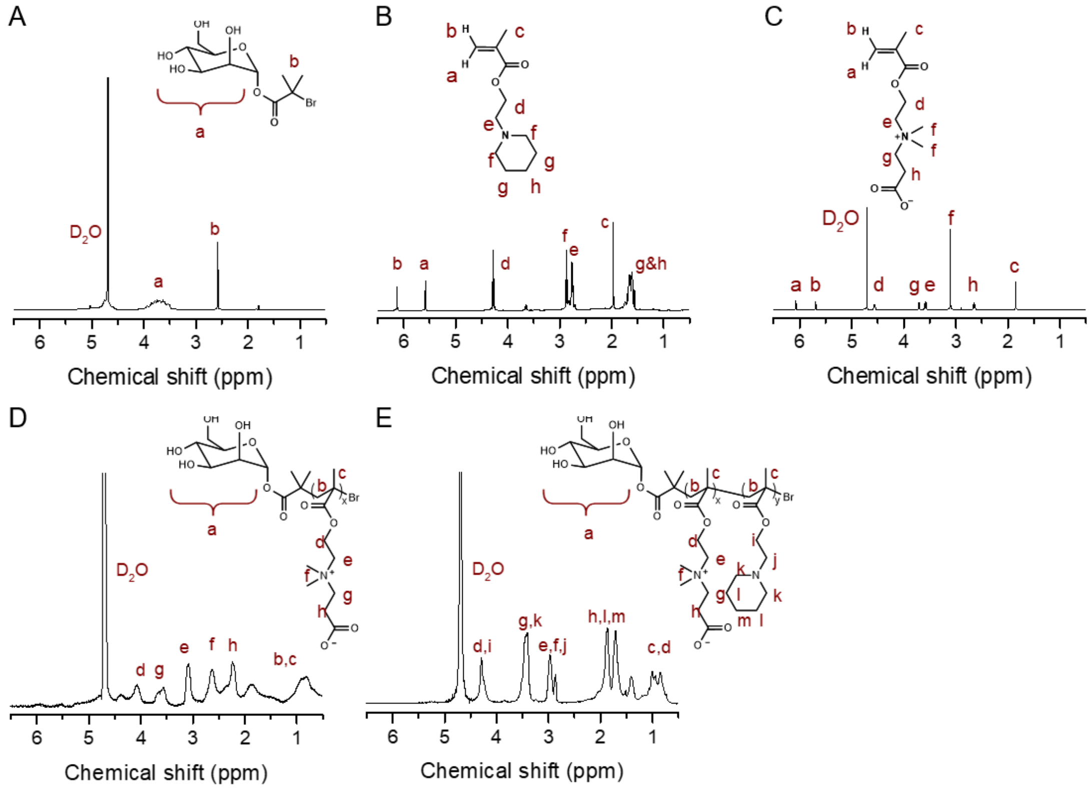

Figure S1. Characterization of synthetic compounds. ^{1}H NMR spectrum of (A) Man-Br, (B) CBMA, (C) N-HEPMA, (D) Man-PCB, and (E) Man-PCB-PHEP.

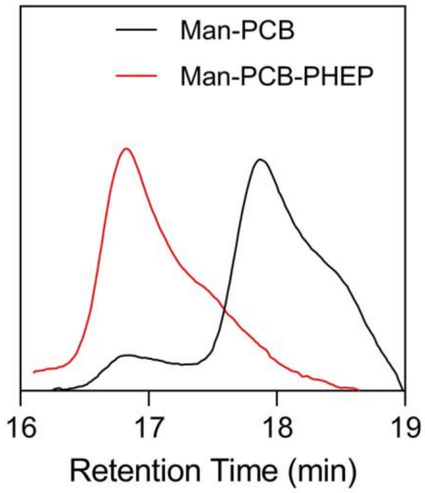

Figure S2. GPC curves of polymer Man-PCB and Man-PCB-PHEP.

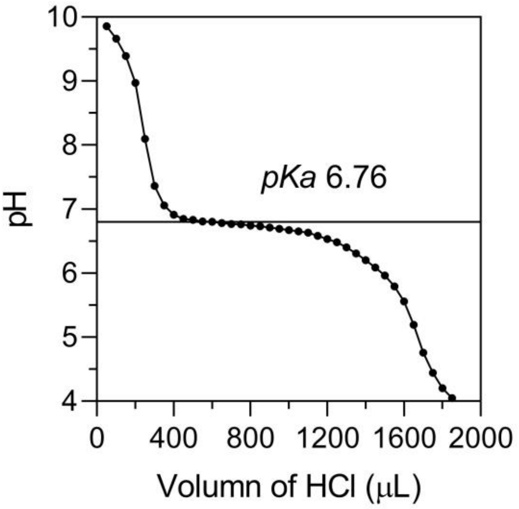

Figure S3. The pH titration curve of Man-PCB-PHEP block copolymer.

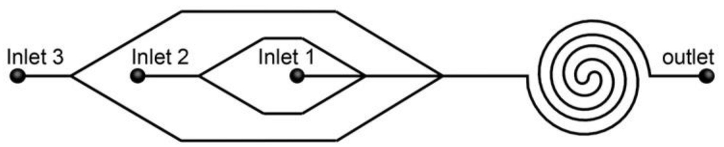

Figure S4. The multi-stage microfluidic chip for producing NCPA.

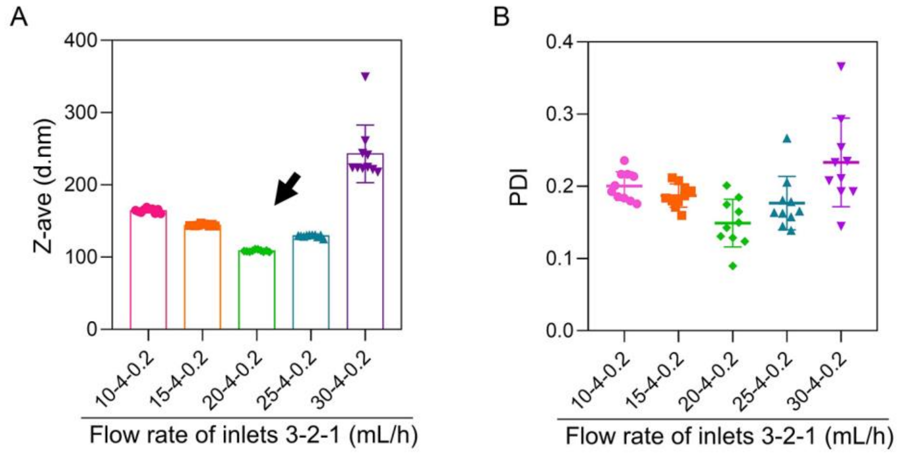

Figure S5. Organic solvent-free microfluidic synthesis of NCPA in one step at different conditions.

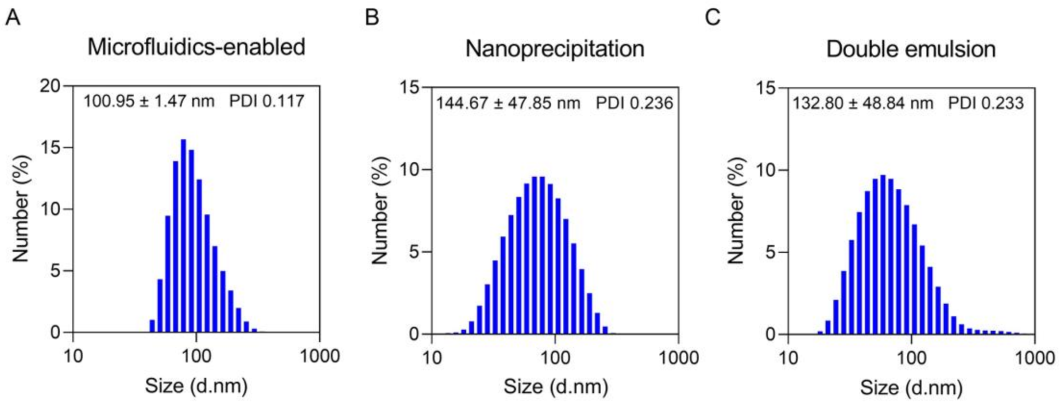

Figure S6. The hydrodynamic diameter of nanoparticles prepared by (A) microfluidics-enabled, (B) nanoprecipitation and (C) double emulsion.

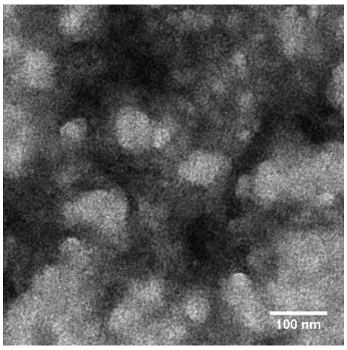

Figure S7. TEM images of NCPA in pH 6.5.

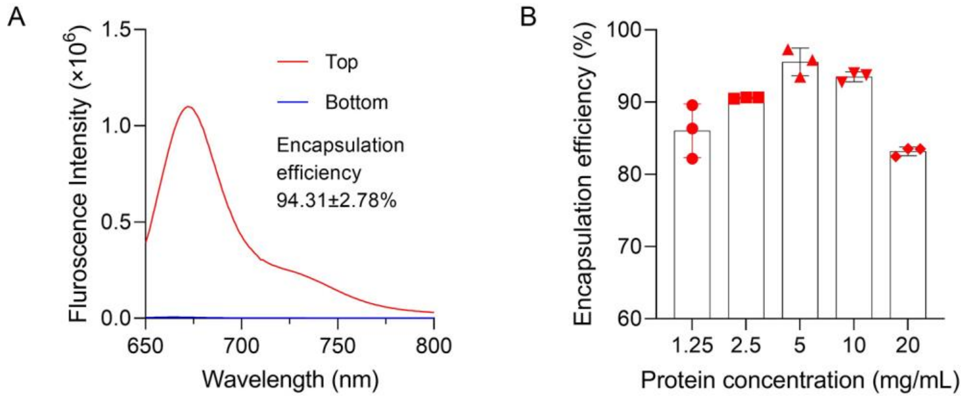

Figure S8. (A) The fluorescence spectrum of CY5 labeled BSA encapsulated in NCPA (Top); (B) The protein entrapment efficiency of NCPA evaluated in different protein concentration.

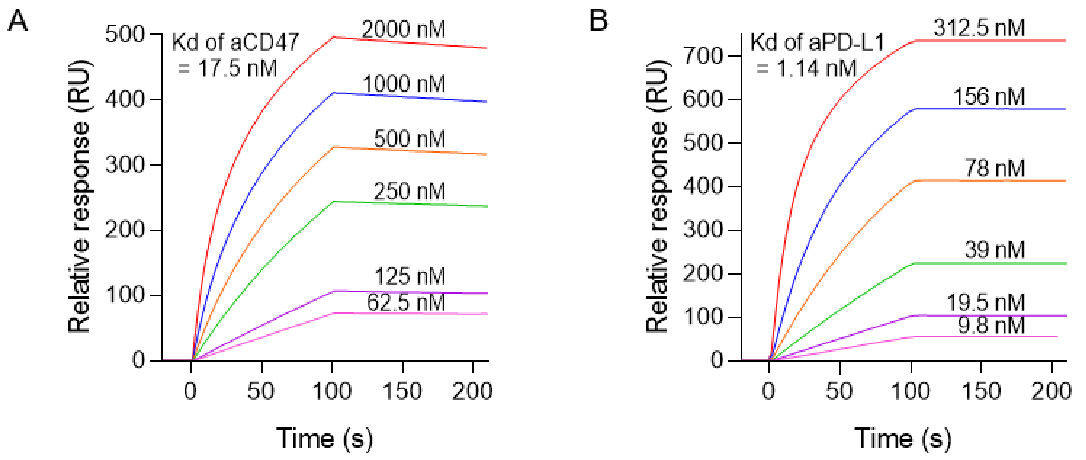

Figure S9. The *in vitro* SPR analysis of (A) CD47 antibody binding to CD47 protein and (B) PD-L1 antibody binding to PD-L1 protein.

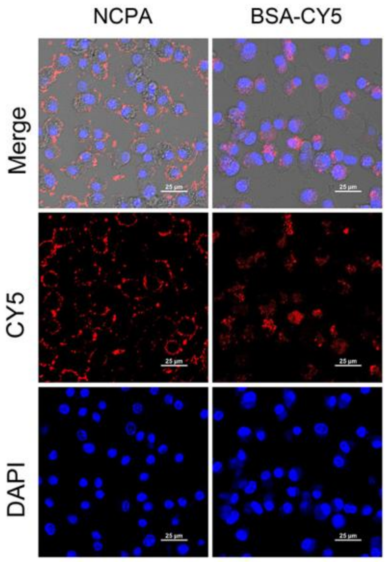

Figure S10. Confocal images of BMDMs (macrophage) cultured with NCPA, the BSA was stained red with CY5.

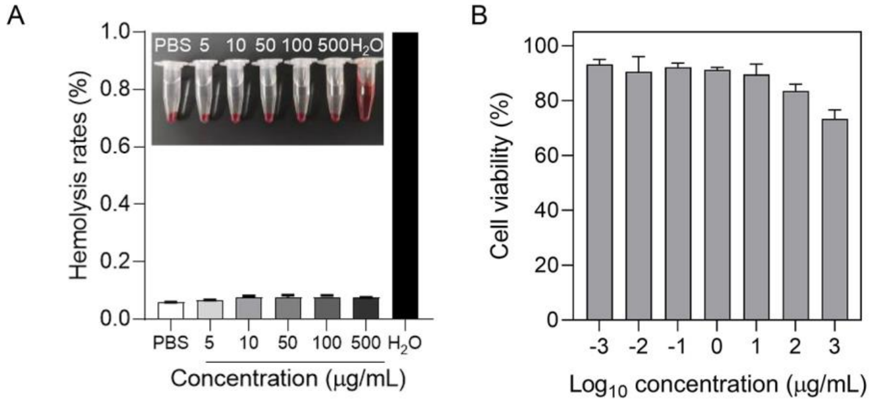

Figure S11. (A) Hemolysis and (B) cytotoxicity tests of NCPA.

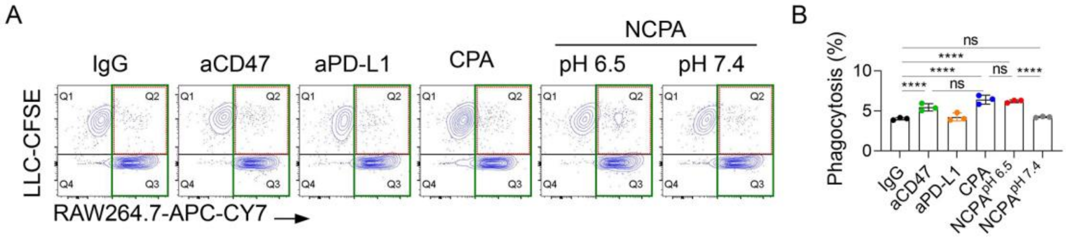

Figure S12. (A) Representative flow cytometry counter diagrams showing the phagocytosis of LLC by RAW264.7 evaluated by tumor spheroid. (B) The phagocytosis was calculated as the percentage of double-positive RAW264.7 among red fluorescence positive RAW264.7, assessed by flow cytometry analysis.

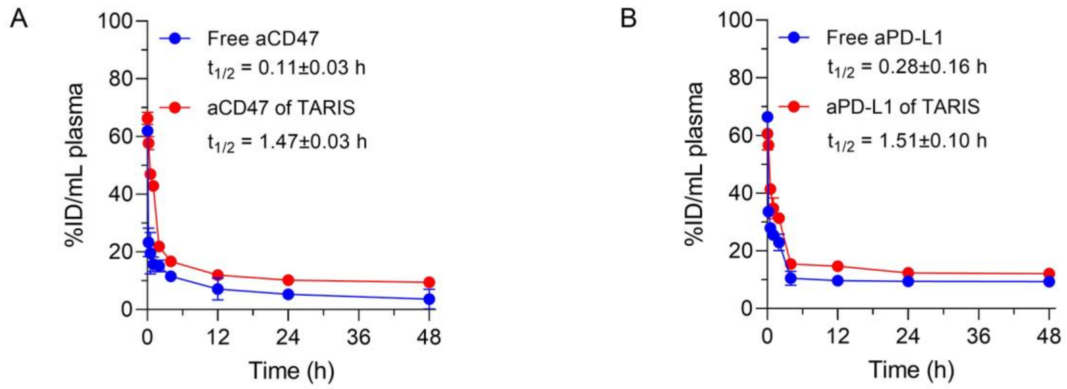

Figure S13. Blood circulation profiles of (A) CD47 antibody and (B) PD-L1 antibody in free condition (blue line) NCPA (red line).

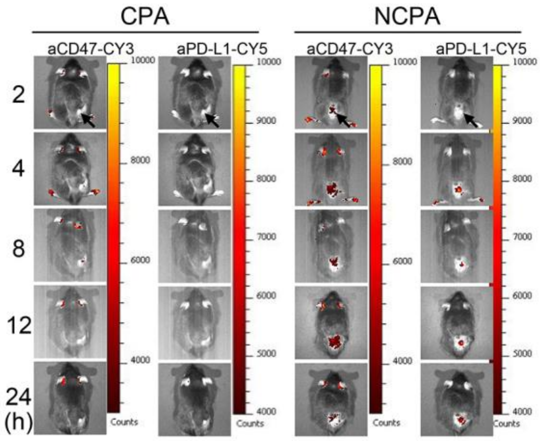

Figure S14. *In vivo* fluorescence images after intravenous injection of fluorescently labeled free antibodies and NCPA.

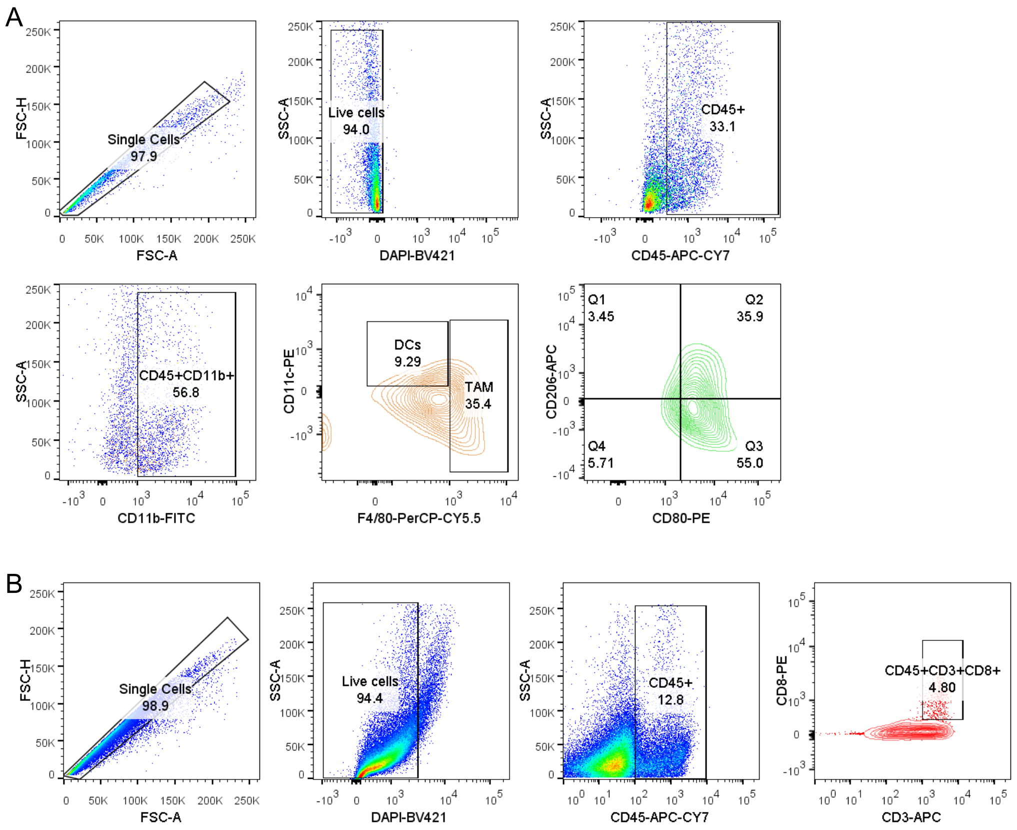

Figure S15. Graphical account for flow cytometry gating strategies of (A) TAM, DCs, and (B) CTLs.

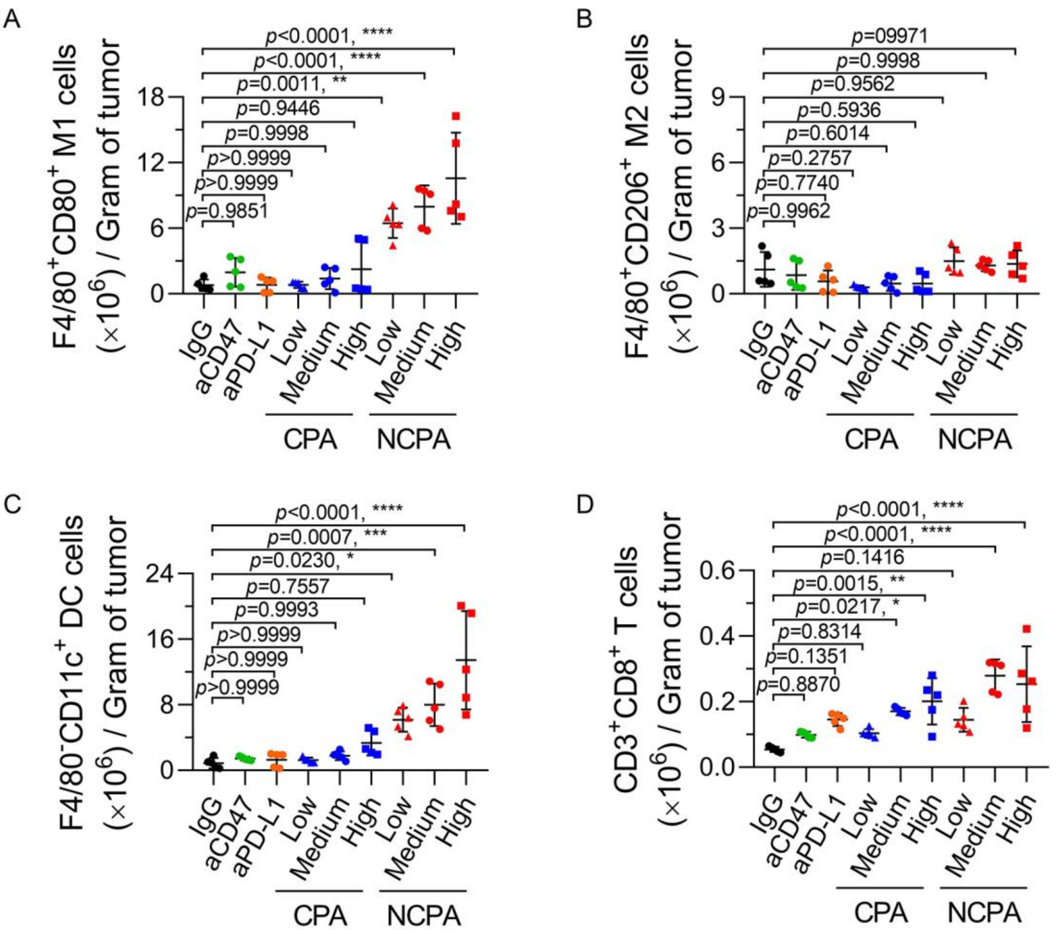

Figure S16. Absolute count measurements of (A) F4/80$^+$CD80$^+$ M1 cells, (B) F4/80$^+$CD206$^+$ M2 cells, (C) F4/80$^-$CD11c$^+$ DC cells and (D) CD3$^+$CD8$^+$ T cells in tumor.

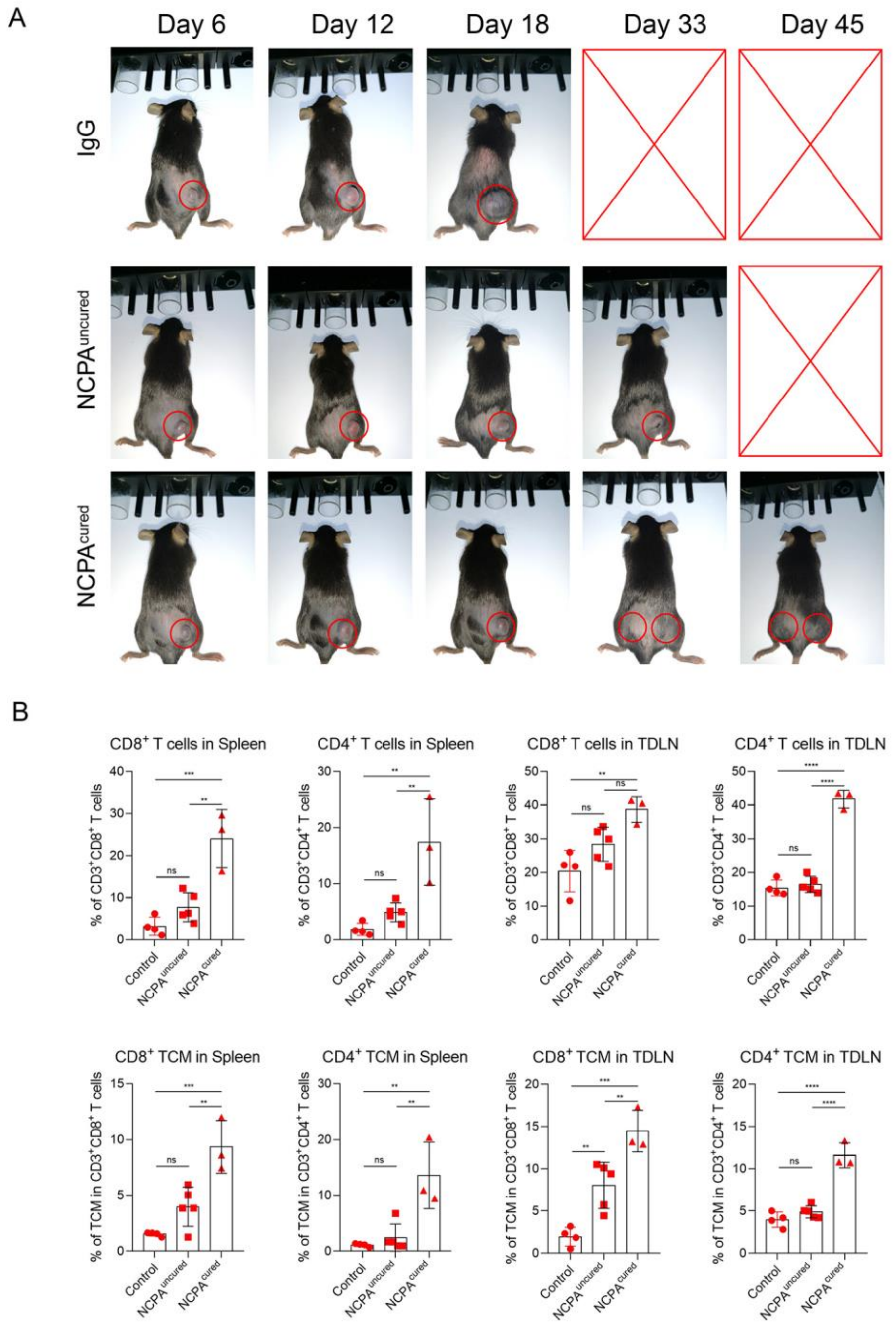

Figure S17. Analysis of immune memory induced by NCPA. (A) Representative images of mice treated with IgG and NCPA, the NCPA cured mice were re-challenged LLC tumor cells (5×10^5/mouse) at the opposite (left flank) at day 35; (B) Flow cytometry analysis of $CD3^+CD8^+$ T cells, $CD3^+CD4^+$ T cells, $CD3^+CD8^+CD44^+CD62L^+$ TCM and $CD3^+CD4^+CD44^+CD62L^+$ TCM in spleen and TDLN.

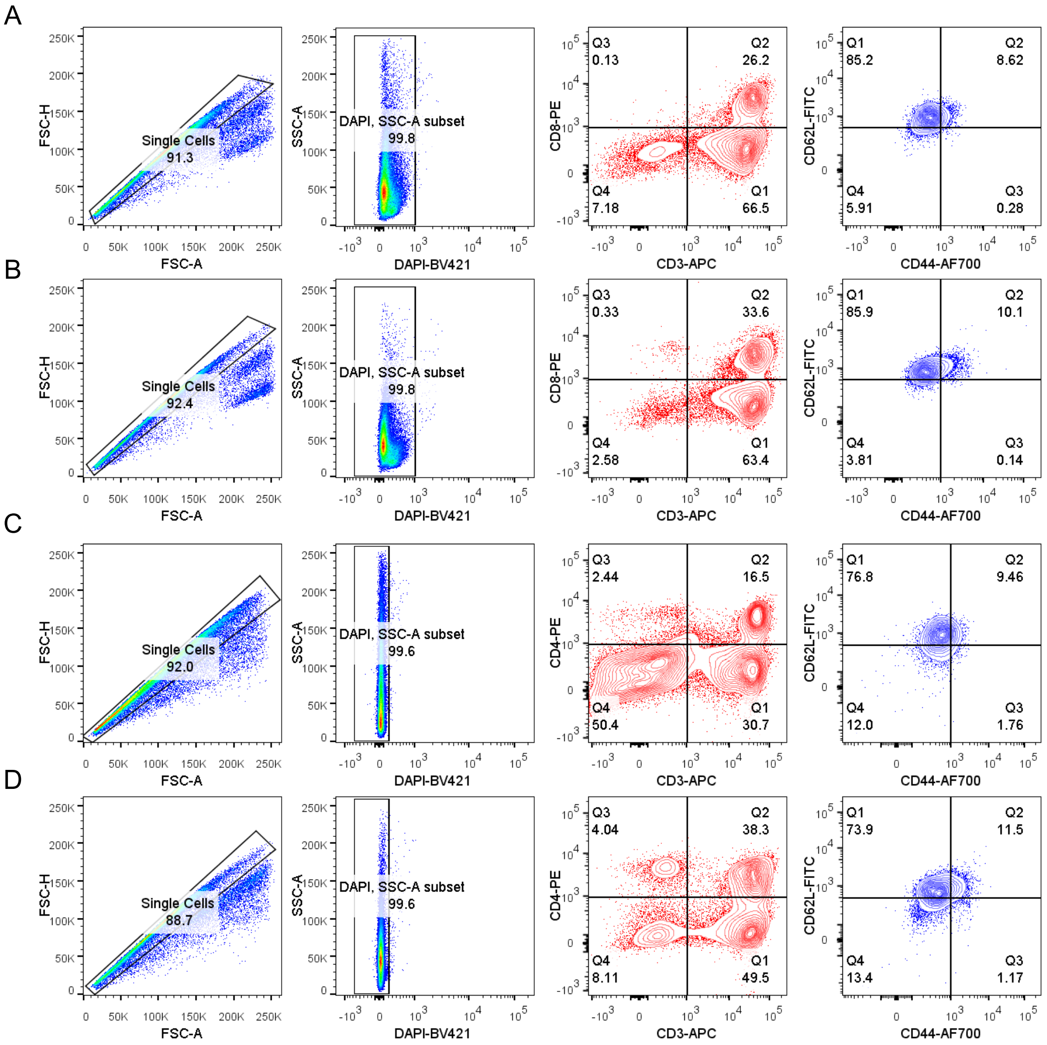

Figure S18. Graphical account for flow cytometry gating strategies of $CD3^+CD8^+CD44^+CD62L^+$ TCM in (A) spleen and (B) TDLN; $CD3^+CD4^+CD44^+CD62L^+$ TCM in (C) spleen and (D) TDLN.

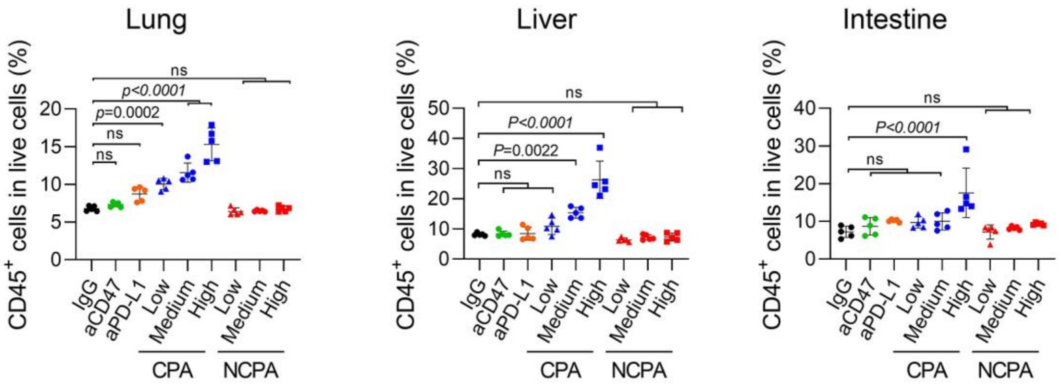

Figure S19. Flowcytometry analysis of activated CD45^{+} lymphocyte infiltration in the lung (A), liver (B), and intestine (C) on day 15 after the indicated treatments.

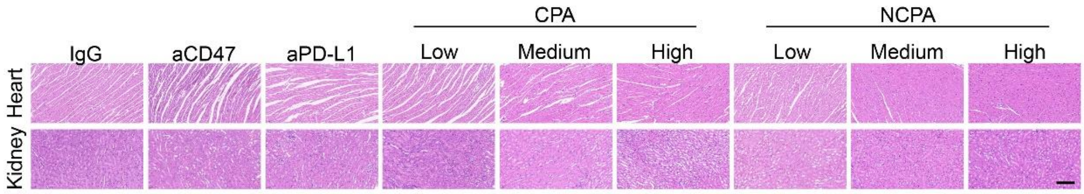

Figure S20. *Ex vivo* pathological H&E staining of the heart and kidney of mice receiving indicated treatments on day 15.

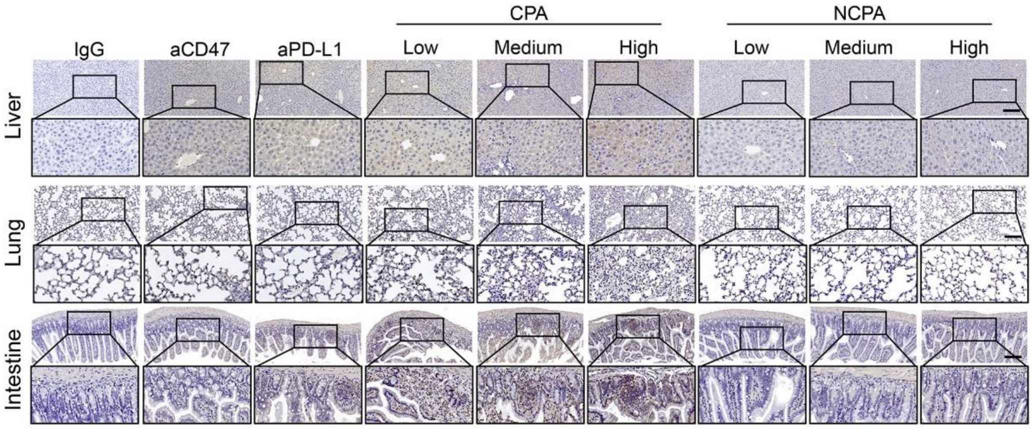

Figure S21. IFN-γ immunohistochemical staining of the liver, lung, and intestine of mice receiving indicated treatments on day 15.

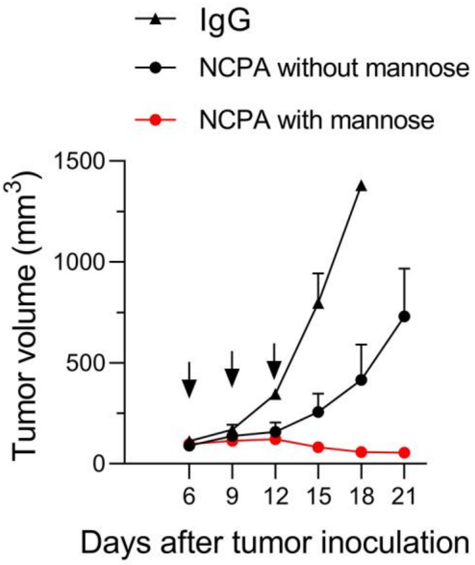

Figure S22. Antitumor growth efficacy of NCPA with or without mannose.

Table S1. Antibodies used in flow cytometry

Target	Fluorophore	Host/Isotype	Clone	Cat#	Supplier
CD45	APC/Cyanine7	Rat/IgG2b, κ	30-F11	103116	
CD11b	FITC	Rat/IgG2b, κ	M1/70	101206	
F4/80	PerCP/Cyanine5.5	Rat/IgG2a, κ	BM8	123127	
CD11c	PE	Armenian Hamster IgG	N418	117307	
CD80	PE	Armenian Hamster IgG	16-10A1	104707	Biolegend
CD206	APC	Rat/IgG2a, κ	C068C2	141708	
CD3	APC	Rat/IgG2b, κ	17A2	100235	
CD4	FITC	Rat/IgG2a, κ	RM4-5	100509	
CD8a	PE	Rat/IgG2a, κ	53-6.7	100707	

Table S2. Antibodies used for immunofluorescence and immunohistochemistry

**Target**	**Sourse/Isotype**	**Cat#**	**Supplier**
IFN-γ	Rabbit/IgG	A12450	Abclonal
CD47	Rabbit/IgG	A1838	
PD-L1	Rabbit/IgG	A 11273	
CD206/MRC1 (E6T5J)	Rabbit/IgG	24595	CST
goat anti-rabbit IgG H&L (Alexa Fluor® 488)		ab150077	Abcam
goat anti-rabbit IgG H&L (Alexa Fluor® 555)		ab150078	
goat anti-rabbit IgG H&L (Alexa Fluor® 647)		ab150079	
goat anti-rabbit IgG H&L (HRP)		ab6721	

Table S3. GPC analysis results of polymer Man-PCB and Man-PCB-PHEP.

Polymer	Mn	Mw	Mz	Mz+1	Mv	PDI
Man-PCB_9	2340	2677	2817	3202	2497	1.14403
Man-PCB_9-$PHEP_{50}$	8636	9871	9441	9968	8954	1.14302

References

[1] S. Rannard, A. Owen, H. Rogers, M. Giardiello, F. Hatton, P. Chambon, S. Auty, A. Dwyer, L. Tatham, *WO2014199174A1*, **2014**.

[2] J. C. Theriot, C. H. Lim, H. Yang, M. D. Ryan, C. B. Musgrave, G. M. Miyake, *Science* **2016**, 352, 1082.

[3] M. Luo, H. Wang, Z. Wang, H. Cai, Z. Lu, Y. Li, M. Du, G. Huang, C. Wang, X. Chen, M. R. Porembka, J. Lea, A. E. Frankel, Y. X. Fu, Z. J. Chen, J. Gao, *Nat. Nanotechnol.* **2017**, 12, 648.

[4] C. W. Shields, M. A. Evans, L. L.-W. Wang, N. Baugh, S. Iyer, D. Wu, Z. Zhao, A. Pusuluri, A. Ukidve, D. C. Pan, S. Mitragotri, *Sci. Adv.* **2020**, 6, eaaz6579.

[5] L. Tang, Y. Zheng, M. B. Melo, L. Mabardi, A. P. Castaño, Y.-Q. Xie, N. Li, S. B. Kudchodkar, H. C. Wong, E. K. Jeng, M. V. Maus, D. J. Irvine, *Nat. Biotechnol.* **2018**, 36, 707.

[6] Y., Chen, D., Zhao, F., Xiao, X., Li, J., Li, Z., Su, X., Jiang. *Adv. Mater.* **2023**, 2209672.
